# Red Spinach Extract Increases Ventilatory Threshold during Graded Exercise Testing

**DOI:** 10.3390/sports5040080

**Published:** 2017-10-16

**Authors:** Angelique N. Moore, Cody T. Haun, Wesley C. Kephart, Angelia M. Holland, Christopher B. Mobley, David D. Pascoe, Michael D. Roberts, Jeffrey S. Martin

**Affiliations:** 1 Department of Cell Biology and Physiology, Edward Via College of Osteopathic Medicine-Auburn Campus, Auburn, AL 36832, USA; amoore@auburn.vcom.edu (A.N.M.); mdr0024@auburn.edu (M.D.R.); 2 School of Kinesiology, Auburn University, Auburn, AL 36849, USA; cth0023@auburn.edu (C.T.H.); wck0007@auburn.edu (W.C.K.); angholland@augusta.edu (A.M.H.); moblecb@auburn.edu (C.B.H.); pascodd@auburn.edu (D.D.P.)

**Keywords:** *Amaranthus dubius*, submaximal, endurance exercise, nitrate, anaerobic threshold

## Abstract

**Background:** We examined the acute effect of a red spinach extract (RSE) (1000 mg dose; ~90 mg nitrate (NO${}_{3}^{-}$)) on performance markers during graded exercise testing (GXT). **Methods:** For this randomized, double-blind, placebo (PBO)-controlled, crossover study, 15 recreationally-active participants (aged 23.1 ± 3.3 years; BMI: 27.2 ± 3.7 kg/m^2^) reported >2 h post-prandial and performed GXT 65–75 min post-RSE or PBO ingestion. Blood samples were collected at baseline (BL), pre-GXT (65–75 min post-ingestion; PRE), and immediately post-GXT (POST). GXT commenced with continuous analysis of expired gases. **Results:** Plasma concentrations of NO${}_{3}^{-}$ increased PRE (+447 ± 294%; *p* < 0.001) and POST (+378 ± 179%; *p* < 0.001) GXT with RSE, but not with PBO (+3 ± 26%, −8 ± 24%, respectively; *p* > 0.05). No effect on circulating nitrite (NO${}_{2}^{-}$) was observed with RSE (+3.3 ± 7.5%, +7.7 ± 11.8% PRE and POST, respectively; *p* > 0.05) or PBO (−0.5 ± 7.9%, −0.2 ± 8.1% PRE and POST, respectively; *p* > 0.05). When compared to PBO, there was a moderate effect of RSE on plasma NO${}_{2}^{-}$ at PRE (g = 0.50 [−0.26, 1.24] and POST g = 0.71 [−0.05, 1.48]). During GXT, VO_2_ at the ventilatory threshold was significantly higher with RSE compared to PBO (+6.1 ± 7.3%; *p* < 0.05), though time-to-exhaustion (−4.0 ± 7.7%; *p* > 0.05) and maximal aerobic power (i.e., VO_2_ peak; −0.8 ± 5.6%; *p* > 0.05) were non-significantly lower with RSE. **Conclusions:** RSE as a nutritional supplement may elicit an ergogenic response by delaying the ventilatory threshold.

## 1. Introduction

Acute and/or sub-chronic exogenous nitrate (NO${}_{3}^{-}$) ingestion has demonstrated potential for improved sub-maximal exercise time-to-exhaustion (TTE) [1], time-trial performance [2,3], and graded exercise testing (GXT) performance [4]. However, others report NO${}_{3}^{-}$ ingestion has limited or no effect on performance outcomes [5,6,7]. Specificity of study designs with varying time-course supplementation (i.e., acute vs. chronic), NO${}_{3}^{-}$ sources, diverse bioactive phytochemicals, or enzymatic changes from enterosalivary circulation may contribute to variable bioavailability and production of nitric oxide (NO) and, thus, influence the observed outcomes [8]. Wylie and colleagues [9] recently characterized responses to acute ingestion of 4.2, 8.4, and 16.8 mmol of NO${}_{3}^{-}$ from beetroot juice. In that study, plasma (all instances of plasma nitrate and nitrite noted in brackets) [NO${}_{3}^{-}$] and [NO${}_{2}^{-}$] increased dose-dependently with peaks observed 2–4 h post-ingestion with the 6.8 mmol dose compared to 1–2 h post-ingestion with the lower doses. Moreover, arterial blood pressure dose-dependently decreased post-ingestion, but a significant effect was observed for the lowest dose (4.2 mmol) at only the 1-h post-ingestion time point, further illustrating a more transient response to lower doses of NO${}_{3}^{-}$. Thus, the timing of NO${}_{3}^{-}$-mediated effects may depend upon the dose delivered.

An acute 2000 mg dose of red spinach extract (RSE) delivering ~180 mg (~2.9 mmol) of NO${}_{3}^{-}$ has been shown to increase plasma [NO${}_{3}^{-}$] and [NO${}_{2}^{-}$] to peaks of 253 μmol/L (ca. a four-fold change) and 0.56 μmol/L (~1.8-fold change), respectively [10]. Interestingly, these peak concentrations are similar to, or exceed, those observed with acute ingestion of relatively higher NO${}_{3}^{-}$ doses from beetroot juice [9,11]. Moreover, with acute RSE ingestion plasma [NO${}_{3}^{-}$] peaked at 1 h, although plasma [NO${}_{2}^{-}$] was highly variable between consecutive 15 min time points with the largest spikes between 30–180 min post-ingestion [10]. Collectively, the data suggest differential pharmacokinetics from those observed with beetroot. The majority of inorganic nitrate supplementation studies regarding exercise performance have utilized beetroot. Remarkably, limited studies have evaluated NO${}_{3}^{-}$ rich leafy vegetable and, more specifically, RSE on exercise performance [12]. Importantly, not only is RSE rich in NO${}_{3}^{-}$, potassium (>10% by weight), and anti-oxidant polyphenols (e.g., amaranthine), but it is also devoid of sugar and oxalates.

Given the aforementioned more transient elevations in plasma [NO${}_{3}^{-}$] and [NO${}_{2}^{-}$] with lower doses of NO${}_{3}^{-}$, as well as previous work illustrating peak plasma [NO${}_{3}^{-}$] occurring between 45–90 min post-ingestion of 2000 mg of this RSE [10], we chose to evaluate the effects on physical performance at 65–75 min post-RSE/placebo (PBO) consumption. This RSE is included in numerous multi-ingredient ‘pre-workout’ and ‘energy’ supplements (as Oxystorm^®^) with doses ranging from ~500–2000 mg. Thus, we chose to use a ‘medial’ dose of 1000 mg to determine whether ingestion of the same RSE nitrate source previously characterized [10] would increase plasma [NO${}_{3}^{-}$] and [NO${}_{2}^{-}$] and affect GXT performance. We hypothesized the acute medial dose of RSE would increase exercise economy and improve oxygen utilization during GXT.

## 2. Methods

### 2.1. Participants

Fifteen (*N* = 15; males = 8, females = 7) participants were recruited through a community advertisement for this crossover, double-blinded study. Participants included were 19–35 years of age, free from metabolic disease and hypertension (SBP < 140 mmHg and DBP < 90 mmHg), recreationally active (>30 min of physical activity at least 3 days/week), and not consuming any ‘supplements’ for >1 month. All procedures were approved by the Auburn University Institutional Review Board and conformed to the standards set by the latest revision of the Declaration of Helsinki.

### 2.2. Study Design

Participants reported for two visits separated by >72 h, before the primary testing session. For each visit, participants were instructed to abstain from exercise and alcohol for 24 h and caffeine for 12 h. Participants were also asked to adhere to a low NO${}_{3}^{-}$ diet for >48 h adapted from the National Heart, Blood, and Lung Institute [13] to reduce the effects of diet on circulating nitrate and nitrate concentrations and to abstain from mouthwash due to its potential to inhibit bio-activation of NO${}_{3}^{-}$ [14] for 24 h prior to visits. Finally, participants were instructed to replicate their 24 h diet prior to visits and report >2 h post-prandial. In order to control for potential diurnal variation, all participants reported for their visits at the same time of day. Some of these data (i.e., participant characteristics) have been reported previously [15].

Upon reporting for each visit, adherence to guidelines was verbally confirmed. Thereafter, participants’ height and weight were measured, a venipuncture was performed (BL), and a dose of RSE or PBO was ingested. At one occasion 65–75-min post-RSE/PBO ingestion, venipuncture was performed (PRE) after which subjects were immediately prepared for GXT. Maximal GXT commenced using the Bruce protocol with analysis of expired gases. At the conclusion of the GXT, a final venipuncture was performed (POST).

### 2.3. Blood Collection and Humoral Nitrate/Nitrite Concentrations

For each venipuncture (BL, PRE and POST), ~4 mL of venous blood was collected from the antecubital space in tubes containing ethylenediaminetetraacetic acid (EDTA) and immediately centrifuged at 4000 g at 4 °C for 5 min. Plasma was then separated in cryotubes and immediately placed in an −80 °C freezer for later batch-processing. Following a thaw at room temperature, plasma samples were deproteinized using a cold ethanol precipitation. Briefly, the ethanol was pre-cooled to 0 °C and 500 μL of each plasma sample was added to 1 mL of the cold ethanol and allowed to let stand for 30 min at 0 °C. Thereafter, samples were vortexed and centrifuged at 14,000 rpm for 5 min. The resultant supernatant was collected for assessment of total plasma [NO${}_{3}^{-}$], [NO${}_{2}^{-}$] (NO_x_), and plasma [NO${}_{2}^{-}$] only via ozone-based chemilluminescence [16,17] using a NO analyzer (Sievers NOA 280i, Boulder, CO, USA). To determine [NO_x_], samples were added to 0.1 M vanadium chloride in 1 M hydrochloric acid refluxing at 95 °C under nitrogen. To determine [NO${}_{2}^{-}$], samples were added to aqueous iodine in 1 M acetic acid refluxing at room temperature under nitrogen. A cooled photomultiplier tube housed in the NOA detected respective reduction to NO and concentrations of NO_x_ and NO${}_{2}^{-}$ were determined by a plotting signal (mV) against a calibration plot. [NO${}_{3}^{-}$] was calculated as [NO_x_] − [NO${}_{2}^{-}$].

### 2.4. Supplementation: Red Spinach Extract and Placebo

A single oral dose of 1000 mg RSE powder (i.e., Oxystorm^®^) or PBO (maltodextrin) in gelatin capsules was consumed with bottled water. Per manufacturer, the RSE is derived from only pure *Amaranthus dubius*, has an herb to extract ratio of 50:1, and contains >9% NO${}_{3}^{-}$ (by HPLC) and >13% potassium (by ICP-MS). The expected ingested dose of nitrate in the RSE was ~90 mg.

At the first visit, ingestion of RSE or PBO was determined at random (flip of coin) and the alternate supplement was ingested at the second visit. Investigators and subjects were un-blinded after completion of data collection and analysis.

### 2.5. Graded Exercise Testing

Participants underwent maximal GXT according to the Bruce protocol, which includes progressive increases in treadmill belt speed and deck incline every 3 min. Expired gases were collected with the subjects standing stationary on the treadmill until stable. Thereafter, participants were allowed to warm up by walking at 2.7 km/h at a 0% incline for 3 min followed immediately by initiation of the protocol (Table 1). GXT continued until participants reached volitional fatigue. Expired gases were continuously analyzed using a TrueMax 2400 metabolic measurement system (ParvoMedics, Sandy, UT, USA) and averaged in 20 s intervals.

**Figure 1:**
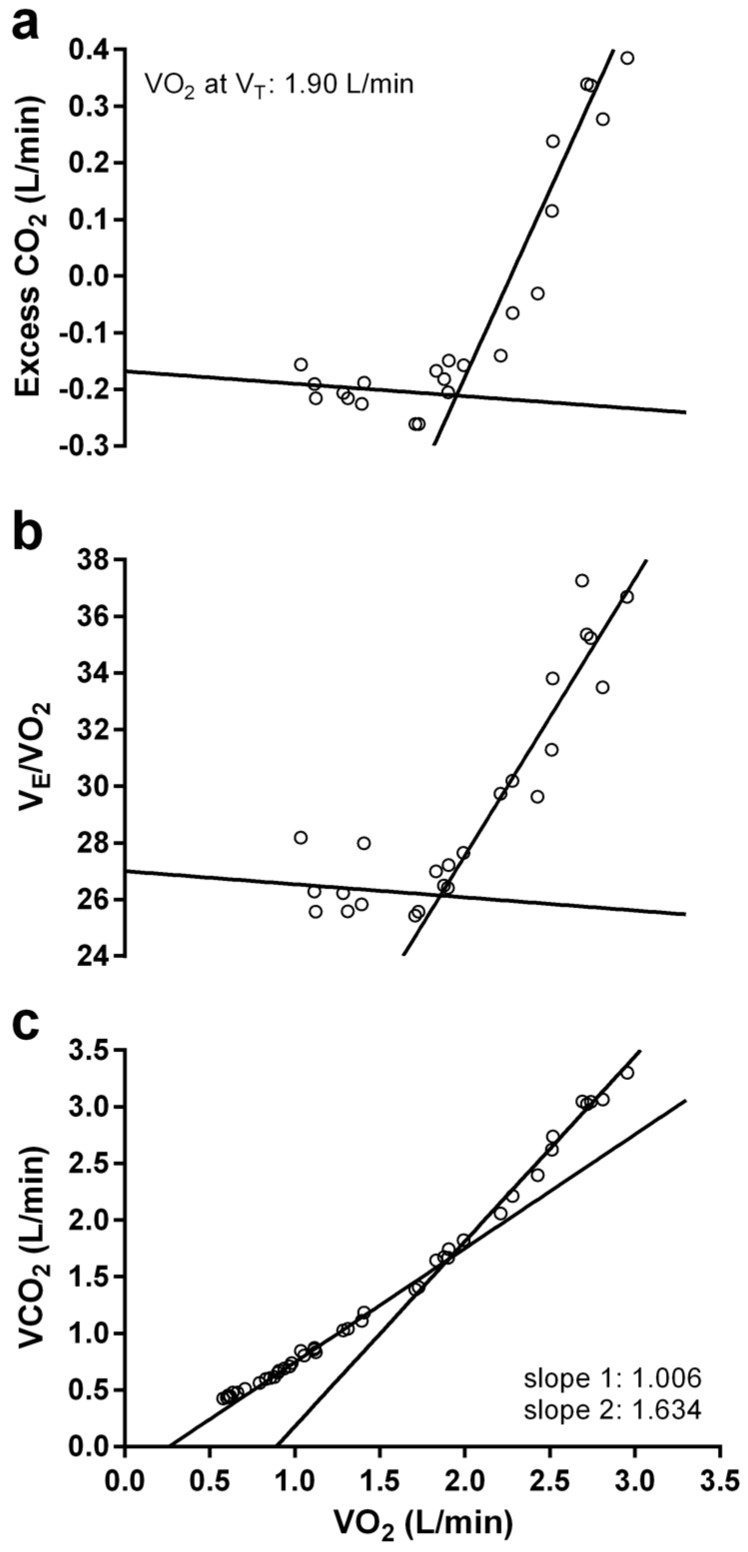
Representative illustration of computer plots showing agreement between the (**a**) excess CO_2_ (ExCO_2_); (**b**) ventilatory equivalent (VEQ); and (**c**) modified v-slope methods for determination of the ventilatory threshold (VT) using expired gas data. Open circles indicate 20 s average expired gas data points and solid lines indicate regression lines fitted to the pre-VT and post-VT segments of the data set.

Peak oxygen consumption rates (VO_2_ peak) were determined using the highest 20 s average observed during the GXT and TTE was recorded as the time at which participants indicated volitional fatigue. The ventilatory threshold (VT) was identified by a computer program (WinBreak 3.7, Epistemic Mindworks, Ames, IA, USA) using multiple parallel methods of determination; excess carbon dioxide (ExCO_2_) [18], ventilatory equivalent ((VEQ), [19]), and modified v-slope [20] methods. ExCO_2_ was calculated as ((VCO_2_^2^/VO_2_)-VCO_2_) and plotted over time with the point at which ExCO_2_ began to increase disproportionately identified as the VT [18]. For the VEQ method, the VT was identified as the first point at which the ventilatory equivalent for O_2_ increased without a concurrent increase in the ventilatory equivalent for CO_2_. Finally, for the modified v-slope method, (1) a plot of VCO_2_ vs. VO_2_ was divided into two regions with each segment being fit with linear regression; (2) the location of the intersection between the two regression lines was calculated where the point dividing the two regions best fit the data by maximizing the ratio of the greatest distance of the intersection point from the single regression line of the data to the mean square error of regression; and (3) it was ensured that the slope of the first regression line was >0.6 and the change in slope from the first regression to the second was >0.1 [21]. For the ExCO_2_ and VEQ methods, a standard algorithm for identifying the break point of two lines was used [22] and for the v-slope method the algorithm proposed by Beaver, Wasserman, and Whipp [20] was used. An illustration of the VT identification methods is presented in Figure 1. An average of the three methods was calculated as VT and the CV between the methods was found to be 3.35%.

### 2.6. Statistical Analysis

All data were tested for normal distribution using the Shapiro-Wilk test. An alpha level of 0.05 was required for statistical significance. Independent *t*-tests were performed to compare subject characteristics. For plasma [NO${}_{3}^{-}$] and [NO${}_{2}^{-}$], a repeated measures two-way ANOVA was employed. When a significant treatment-by-time interaction was observed, within- and between-treatment comparisons were performed using Student’s paired *t*-tests. Hedges’ g was calculated to determine effect sizes with values of 0.5, and 0.8 representing moderate, and large effects, respectively [23]. For plasma [NO${}_{3}^{-}$] and [NO${}_{2}^{-}$] at PRE and POST, g was calculated using the ratio of the respective mean difference from BL in the RSE and PBO conditions to the pooled standard deviation of the differences. For GXT parameters, g was calculated using the ratio of the mean difference between treatment conditions to the standard deviation of the differences. Statistical analyses were performed using IBM SPSS Statistics 24 for Windows (Chicago, IL, USA). Data are reported as mean ± SD and effect sizes as mean (95% confidence interval (lower limit, upper limit)).

## 3. Results

### 3.1. Participants

One participant was excluded due to the discovery of non-compliance with dietary guidelines ([NO${}_{3}^{-}$] >3-fold higher than study average at baseline). Participant characteristics are presented in Table 2. There were no significant differences between male and female participants for age, height, weight, BMI, and VO_2_ peak. Moreover, sex and weight were not found to be significant covariates for any variable analyzed.

### 3.2. Circulating Concentrations of Nitrate and Nitrite

**Figure 2:**
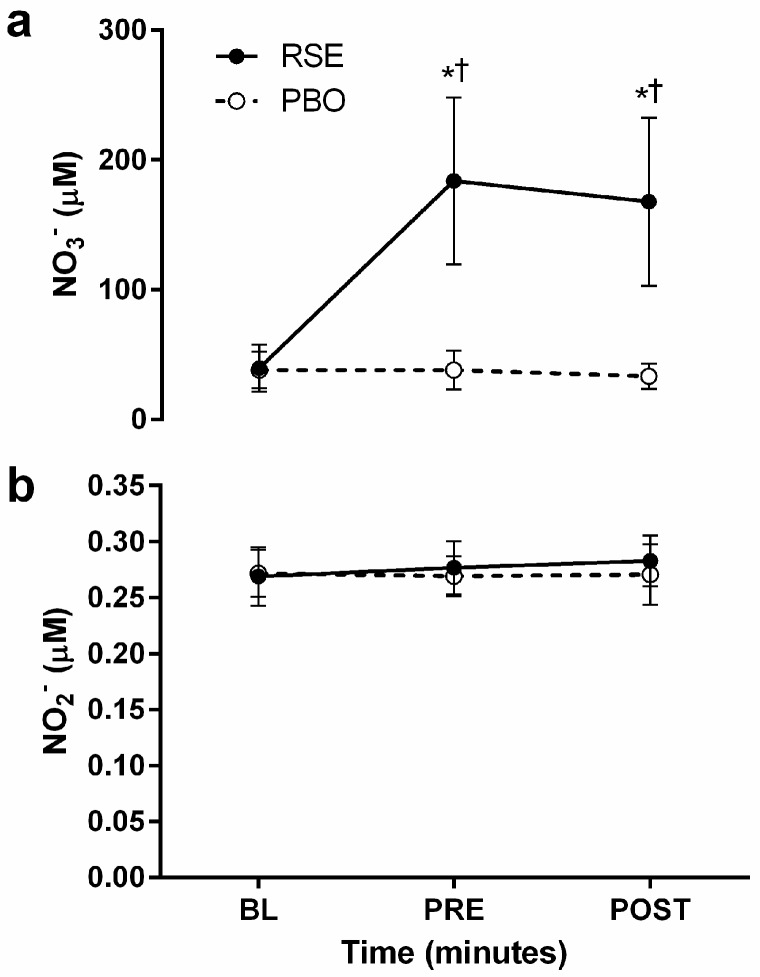
Plasma concentrations of (**a**) nitrate (NO${}_{3}^{-}$) and (**b**) nitrite (NO${}_{2}^{-}$) following ingestion of a red spinach extract (RSE; ●) and placebo (PBO; ○) at baseline (BL), 65–75 min following ingestion (PRE), and immediately following graded exercise testing (POST). Data are presented as mean absolute values in μM concentrations ± SD. When a significant time*treatment interaction was observed, post-hoc tests were performed using Student’s paired *t*-tests. * Significantly different from baseline within conditions (*p* < 0.01). † significantly different between conditions at the same time point (*p* < 0.01), respectively).

Treatment means for [NO${}_{3}^{-}$] and [NO_2_] at BL, PRE, and POST are presented in Figure 2. A time*treatment interaction were observed for [NO${}_{3}^{-}$] (*p* < 0.001 for all). RSE ingestion significantly increased [NO${}_{3}^{-}$] at PRE and POST (*p* < 0.001) and concentrations were significantly higher than PBO at the same time points (*p* < 0.001). Large effect sizes were observed for change in [NO${}_{3}^{-}$] at the PRE (g = 2.95 [1.88, 4.02]) and POST (g = 2.94 [1.88, 4.01]) time points. For [NO${}_{2}^{-}$], no main effects or time*treatment interaction (*p* = 0.278–0.336) was observed. However, moderate effect sizes for the change in [NO${}_{2}^{-}$] were observed at the PRE (g = 0.50 [−0.26, 1.24]) and POST (g = 0.71 [−0.05, 1.48]) time points.

### 3.3. Graded Exercise Testing Performance

**Figure 3:**
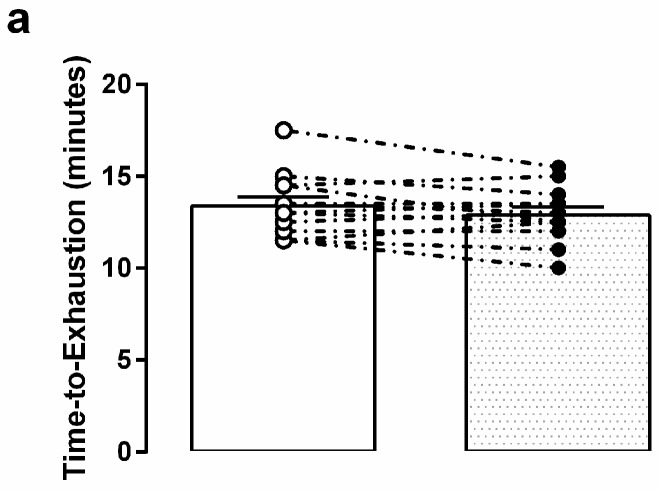

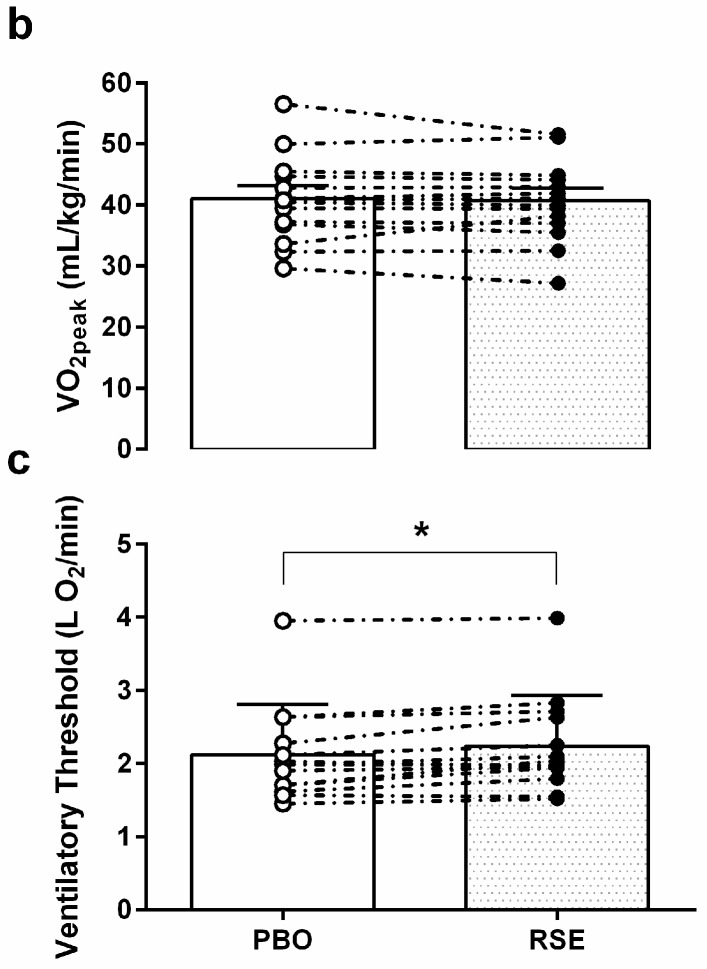
Graded exercise testing (GXT) performance outcomes following acute ingestion of a red spinach extract (RSE; ●) and placebo (PBO; ○). Condition means ± SD, as well as individual values, are presented for (**a**) time-to-exhaustion (in minutes), (**b**) relative VO_2_ peak (in mL/kg/min), and (**c**) observed ventilatory threshold (in L O_2_/min) during the Bruce protocol GXT. Student’s paired *t*-tests were performed to compare performance between conditions. * Significantly different between conditions (*p* < 0.05).

Treatment means and individual values for GXT performance parameters are presented in Figure 3. TTE and VO_2_ peak were not different between conditions (*p* > 0.100; Figure 3a,b) and no moderate or large effect sizes were observed (d < 0.50). VO_2_ at the VT was significantly higher during the RSE condition relative to PBO (*p* = 0.012; Figure 3c) and there was a large effect size (g = 0.73 [0.65, 0.80]). The time at which the VT was observed was not different between conditions (518 ± 110 and 519 ± 97 s for RSE and PBO, respectively; *p* = 0.991), though the percent of VO_2_ peak at which VT was observed was significantly higher with RSE (66.8 ± 10.7 and 63.1 ± 7.6% for RSE and PBO, respectively; *p* = 0.035).

## 4. Discussion

The primary findings are that a single 1000 mg dose of RSE compared to PBO (1) significantly increased plasma [NO${}_{3}^{-}$], but not [NO${}_{2}^{-}$]; and (2) significantly increased the VT during GXT commencing at 65–75 min post-ingestion.

Previously, we reported a three-fold increase in plasma [NO${}_{3}^{-}$] from BL 65–75 min post-ingestion of a single 1000 mg dose of RSE [15]. However, in that study, mean plasma [NO${}_{3}^{-}$] was reported to increase from 12 to 36 µM. Herein, using ozone-based chemiluminescence [NO${}_{3}^{-}$] was found to increase from 40 to 184 µM, representing a 4.6-fold change. The markedly different [NO${}_{3}^{-}$] concentrations and fold-changes likely represent the quantitative limitations associated with [NO${}_{3}^{-}$] and [NO${}_{2}^{-}$] measurement using Griess-based assays [24]. Indeed, the peak plasma [NO${}_{3}^{-}$] response to a single 1000 mg dose of RSE herein was ~73% of that observed in another study utilizing a single 2000 mg dose of RSE [10]. Thus, given the greater sensitivity of the ozone-based chemiluminescence method [17] and a dose-response that appears to be consistent with previous RSE literature, from a quantitative standpoint our current findings are likely more accurate than those previously reported.

Despite utilizing methods with greater sensitivity for detecting plasma [NO${}_{2}^{-}$], no statistically significant effect of RSE ingestion on plasma [NO${}_{2}^{-}$] was observed. Moreover, the moderate effect sizes suggesting an increase in [NO${}_{2}^{-}$] at both the PRE and POST time points with RSE ingestion were not greater than their respective 95% confidence interval. The lack of a significant alteration in plasma [NO${}_{2}^{-}$] with RSE ingestion could be due the ingested NO${}_{3}^{-}$ dose. Following un-blinding, 1000 mg of RSE was diluted in nitrate-free water and tested for [NO${}_{3}^{-}$] using the ozone-based chemiluminescence methods described to better characterize the ingested dose. We found ~11.5% NO${}_{3}^{-}$ by weight, which equates to the delivery of ~115 mg of NO${}_{3}^{-}$. It has been previously shown that acute KNO_3_ supplementation, with a NO${}_{3}^{-}$ dose of twice that given herein, resulted in a 1.4-fold peak increase in plasma [NO${}_{2}^{-}$] at ~1 h post-ingestion [25]. Moreover, in an investigation specific to RSE, a dose of twice that delivered herein resulted in ~1.5–2-fold increases in plasma [NO${}_{2}^{-}$] at 30 and 90 min post-ingestion [10]. The plasma [NO${}_{2}^{-}$] fold-change observed in the present study (~1.1) was considerably less than expected, particularly given that we observed peak plasma [NO${}_{3}^{-}$] levels near 200 μM which have been previously associated with significant (greater than two-fold) increases in plasma [NO${}_{2}^{-}$] with beetroot-derived NO${}_{3}^{-}$ [9]. Thus, the present results suggest that the pharmacokinetics associated with RSE may differ significantly from other exogenous NO${}_{3}^{-}$ sources and that a greater increase in plasma [NO${}_{3}^{-}$] from RSE may be required to affect plasma [NO${}_{2}^{-}$].

Regarding GXT performance, no significant alteration in TTE or VO_2_ peak was observed, though previous reports have indicated that exogenous NO${}_{3}^{-}$ supplementation may actually decrease VO_2_ peak [4], or have little [26] to no effect [27] on these outcomes. However, VT during GXT was significantly higher during the RSE condition, suggesting a delayed onset of significant anaerobic metabolism. Given the acute nature of our study, more immediate and plausible proposed mechanisms by which NO${}_{3}^{-}$ supplementation may influence oxygen uptake and utilization could include a reduction of NO${}_{3}^{-}$ to NO, directly influencing mitochondrial efficiency [28], and vascular tone [8] and/or tissue oxygenation [11,29]. However, the support for these mechanisms hinges on the appearance of NO${}_{2}^{-}$ in the plasma and we did not observe a significant effect of acute RSE ingestion on plasma [NO${}_{2}^{-}$]. Though it is plausible that peak plasma [NO${}_{2}^{-}$] could have occurred later due to the lag time associated with NO${}_{2}^{-}$ appearance [30,31], the point remains that a marked increase of plasma [NO${}_{2}^{-}$] was not observed at time points before or after the GXT protocol.

Previous studies have mostly evaluated exercise performance based upon a pharmacokinetic profile consistent with higher doses of NO${}_{3}^{-}$ that demonstrate peak plasma [NO${}_{2}^{-}$] occurring ~2.5 h post-ingestion. Herein, we evaluated the effects of RSE on exercise performance at a considerably earlier time point (i.e., 65–75 min post-ingestion) due to the aforementioned plasma [NO${}_{3}^{-}$] and [NO${}_{2}^{-}$] responses to lower doses of exogenous NO${}_{3}^{-}$_,_ and specifically RSE. Thus, the timing of exercise performance measures post-ingestion of exogenous NO${}_{3}^{-}$ may warrant further consideration, particularly as it relates to lower doses. In addition, beyond NO${}_{2}^{-}$, it remains possible that other phytochemicals and/or non-elucidated mechanisms contributed to our observations with submaximal exercise performance and RSE.

Though alternative, non-NO${}_{2}^{-}$ mediated mechanisms that could affect VT without changes in VO_2_ peak is beyond the scope of this study, a few points warrant consideration. First, we have previously reported an increase in lower limb resistance vessel reactivity ~1 h following acute ingestion of a 1000 mg dose of RSE [15] that may improve oxygen delivery/utilization in skeletal muscle during exercise. Moreover, RSE is rich in potassium, which is strongly associated with ventilation and may contribute to the exercise induced hyperemic response [32]. Secondly, it is noteworthy that RSE is devoid of sugar (in contrast to beetroot juice), which may affect the metabolic response to exercise. Third, although an antioxidant profile is not exclusive to RSE, some polyphenols are unique in RSE (e.g., amaranthine) and the relative concentrations of constituents can differ markedly from other exogenous NO${}_{3}^{-}$ sources, which may affect exercise performance (e.g., quercetin). However, the ergogenic potential and pharmacodynamics associated with potassium and specific polyphenols should be investigated. Finally, given that we only verbally confirmed adherence with dietary guidelines we cannot rule out the possibility that nutrition in the days leading up to exercise trials affected the observed outcomes.

### Practical Applications

Practically, although we did not observe an alteration in the TTE or VO_2_ peak with GXT, the results presented herein regarding acute RSE ingestions and exercise are intriguing given that the VT is highly associated with the lactate threshold and endurance exercise performance [18]. If the observed alteration in the VT with RSE ingestion is, in fact, associated with significant alteration of the anaerobic threshold, it is plausible that a 1000 mg dose of RSE may improve sustained sub-maximal exercise performance and/or potentiate higher sub-anaerobic training intensities. However, steady-state submaximal exercise was not investigated herein with participants experiencing a range of submaximal efforts for only brief periods (i.e., 3 min). Further investigations to characterize the effects of RSE on prolonged submaximal exercise performance as well as potential mechanisms for action beyond NO${}_{2}^{-}$ are warranted. Future investigations should specifically evaluate the effects of RSE on more precise markers of the anaerobic threshold, including local markers of skeletal muscle metabolism.

## 5. Conclusions

In summary, we report that acute ingestion of 1000 mg of a RSE substantially increases plasma [NO${}_{3}^{-}$], but not [NO${}_{2}^{-}$]. Additionally, despite the relatively low dose of NO${}_{3}^{-}$ from RSE we observed a large effect on the VT compared to PBO (+0.12 ± 0.14 L/min) with VT occurring at a significantly higher relative VO_2_ (+3.6 ± 5.2%). Finally, it should be considered that this study only included ‘recreationally active’ participants and the potential benefit in athletes needs to be determined.

## Figures and Tables

**Table 1:** Graded exercise testing protocol (Bruce protocol).

Stage	Time (min)	Speed (km/h)	Grade (%)
0	−3–0	2.7	0
1	0–3	2.7	10
2	3–6	4.0	12
3	6–9	5.5	14
4	9–12	6.8	16
5	12–15	8.0	18
6	15–18	8.9	20
7	18–21	9.7	22

**Table 2:** Participant characteristics.

Characteristics	Overall (*n* = 14)	Males (*n* = 7)	Females (*n* = 7)
Age, yrs	23.7 ± 3.2	23.5 ± 2.3	23.9 ± 4.1
Height, m	1.72 ± 0.09	1.77 ± 0.08	1.68 ± 0.09
Weight, kg	81.7 ± 17.3	88.1 ± 13.3	75.3 ± 19.4
BMI, kg/m^2^	27.3 ± 3.8	28.2 ± 2.9	26.4 ± 4.6
VO_2_ peak, mL/kg/min	40.9 ± 7.2	44.7 ± 7.3	37.2 ± 4.9

Data are presented as mean ± SD. BMI, body mass index.
